# Clinical Profile and Outcomes of *Pseudomonas aeruginosa* Infection at Maternity and Children Hospital, Buraydah, Qassim Region, Saudi Arabia

**DOI:** 10.3390/healthcare14203449

**Published:** 2026-10-15

**Authors:** Zouhd Alhabeeb, Anwar Alsakaker, Joud Alfayez, Maha Alharbi, Daniya Alraqibah, Ghadah Alammari, Yousif A. Alosaily, Ibrahim A. Algosi, Faisal A. Alothman, Bader M. Alonazie, Khaled S. Almuhaisen, Mohammed S. Anaam, Faris S. Alnezary, Abrar K. Thabit, Zikria Saleem, Masaad S. Almutairi

**Affiliations:** 1 Department of Pharmacy Practice, College of Pharmacy, Qassim University, Buraydah 51452, Saudi Arabia; 2 Maternity and Children’s Hospital, Qassim Health Cluster, Buraydah 52384, Saudi Arabia; 3 Department of Pharmacy Practice, College of Pharmacy, Taibah University, Madinah 42353, Saudi Arabia; 4 Department of Pharmacy Practice, Faculty of Pharmacy, King Abdulaziz University, Jeddah 22254, Saudi Arabia

**Keywords:** antibiotics, antibiotic resistance, *Pseudomonas aeruginosa*, multidrug-resistant (MDR), antimicrobial susceptibility, bloodstream infection, mortality

## Abstract

Background/Objectives: *Pseudomonas aeruginosa* is a major cause of healthcare-associated infections with increasing antimicrobial resistance. This study investigated its clinical characteristics, antimicrobial susceptibility patterns, and factors associated with in-hospital mortality. Materials and Methods: This single-center retrospective cohort study was conducted at Maternity and Children Hospital, Buraydah, Qassim Region, Saudi Arabia, from January 2022 to January 2025. Medical records of patients with clinically confirmed *P. aeruginosa* infection were reviewed. Demographic, clinical, microbiological, antimicrobial susceptibility, and outcome data were collected. Statistical analyses were performed using SPSS version 22 and R version 4.4.1. Results: Among 198 patients, 62.1% were children aged 1–10 years, and 24.7% were admitted to the neonatal intensive care unit (NICU)/neonatology ward. Respiratory tract infection was the most common diagnosis (22.7%). Among the tested isolates, susceptibility was highest to amikacin (170/182, 93.4%), gentamicin (142/159, 89.3%), piperacillin/tazobactam (155/183, 84.7%), cefepime (65/77, 84.4%), imipenem (146/176, 83.0%), meropenem (62/75, 82.7%), and ceftazidime (145/181, 80.1%). Agents with intrinsic resistance or limited/non-applicable activity against *P. aeruginosa* were not interpreted as evidence of acquired antimicrobial resistance. Firth multivariable logistic regression identified NICU/neonatology admission (adjusted odds ratio [AOR] = 7.16, 95% CI: 2.77–19.87; *p* < 0.001) and bloodstream infection (AOR = 13.20, 95% CI: 2.25–80.44; *p* = 0.005) as independent predictors of in-hospital mortality. Conclusions: *Pseudomonas aeruginosa* demonstrated substantial antimicrobial resistance, although several clinically relevant antipseudomonal agents, particularly amikacin, retained high activity. NICU/neonatology admission and bloodstream infection were associated with increased mortality, highlighting the importance of early recognition and continued local antimicrobial resistance surveillance.

## 1. Introduction

*Pseudomonas aeruginosa* is an opportunistic Gram-negative pathogen that causes a broad range of community- and healthcare-associated infections, including pneumonia, bloodstream, urinary tract, and wound infections, particularly among hospitalized and vulnerable patients [1,2]. Its ability to develop multiple antimicrobial resistance mechanisms makes infections difficult to treat [1,2,3,4,5,6,7]. Neonates and infants are particularly vulnerable because of immature immune function, prolonged hospitalization, invasive procedures, and frequent exposure to broad-spectrum antibiotics, with *P. aeruginosa* being an important cause of healthcare-associated infections and sepsis in neonatal intensive care settings [8,9,10,11,12].

Antimicrobial resistance in *P. aeruginosa* has substantially reduced available therapeutic options, with resistance reported across several major antimicrobial classes, including carbapenems, cephalosporins, aminoglycosides, and fluoroquinolones [9]. According to the annual report from the European Centre for Disease Prevention and Control (ECDC), 18.7% of the *P. aeruginosa* isolates were found to be carbapenem-resistant, while 13.4% were resistant to three or more antimicrobial classes [13,14]. Isolates are classified using internationally accepted definitions: multidrug-resistant (MDR) isolates are non-susceptible to at least one agent in three or more antimicrobial categories; extensively drug-resistant (XDR) isolates are non-susceptible to at least one agent in all but two or fewer categories (i.e., bacterial isolates remain susceptible to only one or two categories); and pandrug-resistant (PDR) isolates are non-susceptible to all agents in all categories [9,15,16,17].

Management of MDR *P. aeruginosa* infection requires timely detection of individuals who have an increased risk of infection. Among the common risk factors for such infections are prior *P. aeruginosa* infection, prolonged hospital stays, intensive care unit (ICU) admissions, presence of chronic illnesses, and the use of invasive medical equipment [18,19,20,21]. In the United States, MDR *P. aeruginosa* is responsible for 13–19% of hospital-acquired infections annually [22]. In Saudi Arabia, recent reports have shown a growing concern regarding antimicrobial resistance in *P. aeruginosa*, particularly among hospitalized patients. A study conducted at King Fahad Medical City in Riyadh analyzed 3579 *P. aeruginosa* isolates collected between 2019 and 2021, revealing that 9.7% were MDR and 11.8% were XDR [16]. Additionally, a recent national multicenter surveillance study collected 185 *P. aeruginosa* isolates from seven hospitals across seven regions of Saudi Arabia during 2022–2023 and reported that all isolates met the study-defined criteria for multidrug resistance; 22.2% were classified as difficult-to-treat (DTR) and 5.4% as PDR [23]. These findings underscore the escalating threat of resistant *P. aeruginosa* strains in Saudi healthcare settings, particularly in intensive care units [24].

Despite increasing evidence of antimicrobial resistance in *P. aeruginosa* in Saudi Arabia, limited data are available on the combined clinical characteristics, infection sites, antimicrobial susceptibility patterns, and mortality-associated factors of *P. aeruginosa* infections specifically within maternity and pediatric hospital populations in the Qassim region. The findings of this study may provide locally relevant evidence to support antimicrobial stewardship, infection prevention, and the identification and management of high-risk patients.

## 2. Methods

### 2.1. Study Design

This single-center retrospective cohort study was conducted at Buraydah Maternity and Children Hospital, Buraydah, a tertiary care hospital in the Qassim region of Saudi Arabia. The hospital specializes in providing medical care to pediatric and maternal patients. The data comprised patients who were diagnosed between January 2022 and January 2025. Data were gathered from the hospital’s electronic medical records. The study population included both pediatric and maternal patients, and only the patients who had confirmed *P. aeruginosa* infection during the study period were included. Only patients with clinically confirmed *P. aeruginosa* infection were included; culture positivity was interpreted alongside the documented clinical diagnosis and signs of infection. The diagnosis was based on the treating physician’s documented clinical assessment together with relevant microbiological findings. Patients with colonization without clinical evidence of infection were excluded, including surveillance cultures obtained for colonization screening. For patients with multiple positive cultures during the same hospitalization, only the first clinically relevant *P. aeruginosa* isolate was included, regardless of subsequent susceptibility profiles, to avoid duplicate observations.

Patients who were infected with other pathogens or diagnosed outside the specified time period were excluded. Ethical approval was obtained from the Qassim Province Regional Research Ethics Committee, and the study was registered and assigned IRB # 607/46/3230. Informed consent was waived by the Institutional Review Board due to the retrospective nature of the study and the use of de-identified patient records. To maintain patient confidentiality, all data were anonymised, and access was restricted to authorized research team members.

The unit of analysis was the individual patient, with one clinically relevant *P. aeruginosa* isolate representing each infection episode. Each record included the corresponding *P. aeruginosa* isolate and its antimicrobial susceptibility results. Thus, the clinical and outcome analyses were conducted at the patient level, while antimicrobial susceptibility analyses were based on the corresponding isolate-level susceptibility results.

### 2.2. Data Collection

The variables collected encompassed four main domains:

Demographic:

The collected data included patient age, sex, and the hospital ward to which each patient was admitted. The study population comprised both pediatric and maternal adult patients. Pediatric patients were further categorized into infants (<1 year), children (1–10 years), and adolescents (11–18 years). Patients were admitted to a variety of hospital units, including the chemotherapy unit, emergency department, general surgery clinic, ICU, neonatology unit, pediatric specialty unit, PICU, and pediatric cardiac surgery unit. Additional combined units that treated both pediatric and female patients were also included, such as pediatric urology surgery, orthopedics, ENT, infectious diseases, pulmonology, pediatric emergency, pediatric day care, female day care, renal dialysis, urogynecology, the high-risk pregnancy unit, and oncology. Data on MDR status were also collected.

Clinical characteristics:

The data included documented diagnosis, such as bloodstream infections (e.g., bacteremia, sepsis, septic shock), central nervous system infections (e.g., meningitis, encephalitis, brain abscess), ENT infections (otitis media and otitis externa), eye infections (e.g., conjunctivitis), ethylmalonic encephalopathy, intra-abdominal infections (e.g., appendicitis, colitis, infected peptic ulcers, abdominal abscesses), respiratory tract infections (pneumonia, bronchitis, upper and lower respiratory infections), skin and soft tissue infections (cellulitis, abscesses, and infected wounds), urinary tract infections (cystitis, pyelonephritis), and other conditions such as bone and joint infections (osteomyelitis, septic arthritis). The anatomical site of infection was recorded (e.g., abdomen, blood, eyes and ears, respiratory tract, skin, urinary tract, wounds, and other locations such as brain, bone, subdural fluid collections, vulva, and pus from central operative wounds).

Diagnosis referred to the clinically documented infection diagnosis, site of infection referred to the anatomical site of infection, and the type of sample referred to the type of clinical specimen from which *P. aeruginosa* was isolated. These variables were recorded independently; therefore, their frequencies could differ because the clinical diagnosis, anatomical infection site, and type of sample were not necessarily identical for every patient.

Risk factors and comorbidities:

The data also documented comorbidities, including cardiovascular diseases, respiratory diseases, kidney diseases, liver diseases, neurological disorders, immunological conditions, malignancies and others. Risk factors such as previous antibiotic therapy, ICU admission, colonisation, immunocompromised state, previous surgery, haemodialysis, tracheostomy, mechanical ventilation, and catheterisation were also recorded.

Microbiological data:

The sources of *P. aeruginosa* isolates included blood, cerebrospinal fluid (CSF), ear and eye swabs, pus, tracheal aspirates, sputum, urine, and other clinical specimens, such as wound swabs, abscesses, bite wounds, catheter tips, and tubing. Each isolate’s culture and sensitivity results were reported as susceptible, intermediate, or resistant. Clinical outcomes were recorded as Alive or Dead.

### 2.3. Antimicrobial Susceptibility Testing

The susceptibility testing covered a wide range of antibiotics from various pharmacological groups. Among the fluoroquinolones tested were ciprofloxacin, levofloxacin, and a combined group termed norfloxacin/ciprofloxacin. Cephalosporins included a number of generations: first-generation (cephalexin, cephalothin), second-generation (cefuroxime, cefoxitin), third-generation (ceftazidime, ceftriaxone, cefotaxime, ceftizoxime, cefataxine, cefime), and fourth-generation (cefepime). Aminoglycosides included gentamicin and amikacin. Sulfonamide-like agents included trimethoprim, sulfamethoxazole, the combination trimethoprim–sulfamethoxazole, and cotrimoxazole. Penicillins tested were ampicillin, amoxicillin, and piperacillin, along with beta-lactam/beta-lactamase inhibitor combinations such as piperacillin–tazobactam, amoxicillin–clavulanic acid, and amoxicillin–sulbactam. Further classes of antibiotics tested included carbapenems (imipenem, meropenem, and ertapenem), tetracyclines (tetracycline and tigecycline), and other antibiotics, including erythromycin (a macrolide), vancomycin (a glycopeptide), chloramphenicol (an amphenicol), colistin (a polymyxin), and aztreonam (a monobactam). Antimicrobial susceptibility testing (AST) was performed using the MicroScan automated system (Beckman Coulter, Brea, CA, USA), which provides minimum inhibitory concentration (MIC) results. *P. aeruginosa* was identified using routine automated microbiological identification procedures. Susceptibility results were interpreted according to the applicable Clinical and Laboratory Standards Institute (CLSI) breakpoint criteria. Standard laboratory quality-control procedures were followed using recommended reference strains. The same AST platform and interpretive standards were used throughout the study period (2022–2025), with no documented changes in testing methodology or breakpoint criteria. Agents with intrinsic resistance or limited/non-applicable activity against *P. aeruginosa* were not interpreted as evidence of acquired antimicrobial resistance.

Isolates were classified according to internationally accepted definitions of multidrug-resistant (MDR), extensively drug-resistant (XDR), and pandrug-resistant (PDR) phenotypes. MDR was defined as acquired non-susceptibility to at least one agent in three or more antimicrobial categories. XDR was defined as non-susceptibility to at least one agent in all but two or fewer antimicrobial categories, meaning that the isolate remained susceptible to agents in only one or two categories. PDR was defined as non-susceptibility to all agents in all antimicrobial categories. For the purposes of MDR classification in the present study, only resistant results were considered; intermediate results were not considered resistant. Isolates meeting this criterion were classified as MDR.

### 2.4. Statistical Analysis

All statistical analyses were performed using R software, Version 4.4.1 (R Foundation for Statistical Computing, Vienna, Austria) and IBM Statistical Package for the Social Sciences (SPSS) Statistics, version 22 (International Business Machines Corporation, Armonk, NY, USA). Regression analyses were performed using R, while descriptive analyses were performed using SPSS. The dataset was reviewed for completeness before analysis. No missing data were identified for the demographic, clinical, or outcome variables included in the analyses. Descriptive statistics were used to summarize the demographic, clinical, microbiological, and antimicrobial susceptibility characteristics of patients with *Pseudomonas aeruginosa* infection. Categorical variables were presented as frequencies and percentages. Comparisons between categorical variables and clinical outcome were performed using Pearson’s chi-square test or the Monte Carlo Fisher–Freeman–Halton exact test, as appropriate. Antimicrobial susceptibility results were summarized as the numbers and percentages of susceptible, resistant, and intermediate isolates for each antimicrobial agent. The primary outcome was in-hospital mortality, analyzed as a binary variable (dead = 1, alive = 0). Univariable analyses were performed to evaluate the association between predictor and mortality. Crude (unadjusted) odds ratios (UORs) with 95% confidence intervals (95% CIs) were calculated for each predictor using binary logistic regression. Candidate predictors for multivariable modelling were identified based on their clinical relevance. Because the number of mortality events was relatively small and some predictors contained sparse data, Firth penalized logistic regression was used for the multivariable analysis to reduce small-sample bias. Because only 25 mortality events occurred and some predictors contained sparse data, not all potential predictors were entered simultaneously to minimize over-parameterization, overfitting, and instability of estimates. Firth penalized logistic regression was therefore used for the multivariable analysis to reduce small-sample bias [25]. The final model was restricted to three clinically relevant predictors: NICU/neonatology ward admission, bloodstream infection, and blood specimen. Although bloodstream infection and blood specimen were clinically related, they represented distinct variables, with bloodstream infection reflecting the documented clinical diagnosis and blood specimen representing the microbiological specimen source. Multicollinearity was assessed using variance inflation factors (VIFs), with values ranging from 1.11 to 4.34, indicating no serious multicollinearity among the predictors included in the final model. Adjusted odds ratios (AORs), 95% CIs, and two-sided *p*-values were reported. During univariable analyses, when cell counts were small or zero, a 0.5 continuity correction was applied to allow estimation of odds ratios and 95% CIs. Given the multiple comparisons performed, the results were interpreted cautiously, with emphasis on the magnitude and clinical relevance of the observed associations. Statistical significance was defined as a two-sided *p*-value < 0.05.

## 3. Results

In total, 198 patients with culture-confirmed *Pseudomonas aeruginosa* infection were included in this study. Males accounted for 54.0% (107/198) of cases, while females represented 46.0% (91/198). The study population was predominantly pediatric, with children aged 1–10 years comprising the largest age group (62.1%), followed by infants aged <1 year (12.1%), whereas adults accounted for only 15.1% of the cohort. The NICU/neonatology ward had the highest number of admissions (24.7%), followed by the Pediatrics Specialty ward (16.7%) and the ICU (13.1%). Respiratory tract infection was the most frequent diagnosis (22.7%), followed by urinary tract infection (20.2%), bloodstream infection (17.7%), and skin and soft tissue infection (17.2%). Consistent with these findings, the respiratory tract (22.7%), skin and soft tissue (21.2%), urinary tract (19.7%), and bloodstream (17.2%) were the most common infection sites. Pus was the most frequently collected clinical specimen (30.3%), followed by tracheal aspirates (19.2%), urine (19.2%), and blood (15.2%). Overall, 82 (41.4%) isolates were classified as multidrug-resistant (MDR), while 116 (58.6%) were non-MDR, indicating that the majority of *P. aeruginosa* isolates remained non-MDR. Overall, 173 patients (87.4%) were alive, 25 (12.6%) died during hospitalization (Table 1).

### 3.1. Comparison of Baseline Characteristics Across Clinical Outcomes

Among the 198 patients included in the analysis, 173 (87.4%) were alive and 25 (12.6%) died. Mortality was significantly associated with the ward of admission (*p* = 0.002), diagnosis (*p* < 0.001), and site of infection (*p* = 0.001). Most deaths occurred among patients admitted to the NICU/neonatology ward (68.0%) and those with bloodstream infections (52.0%) (Table 2).

### 3.2. Antimicrobial Susceptibility Profile of Pseudomonas aeruginosa

**Figure 1:**
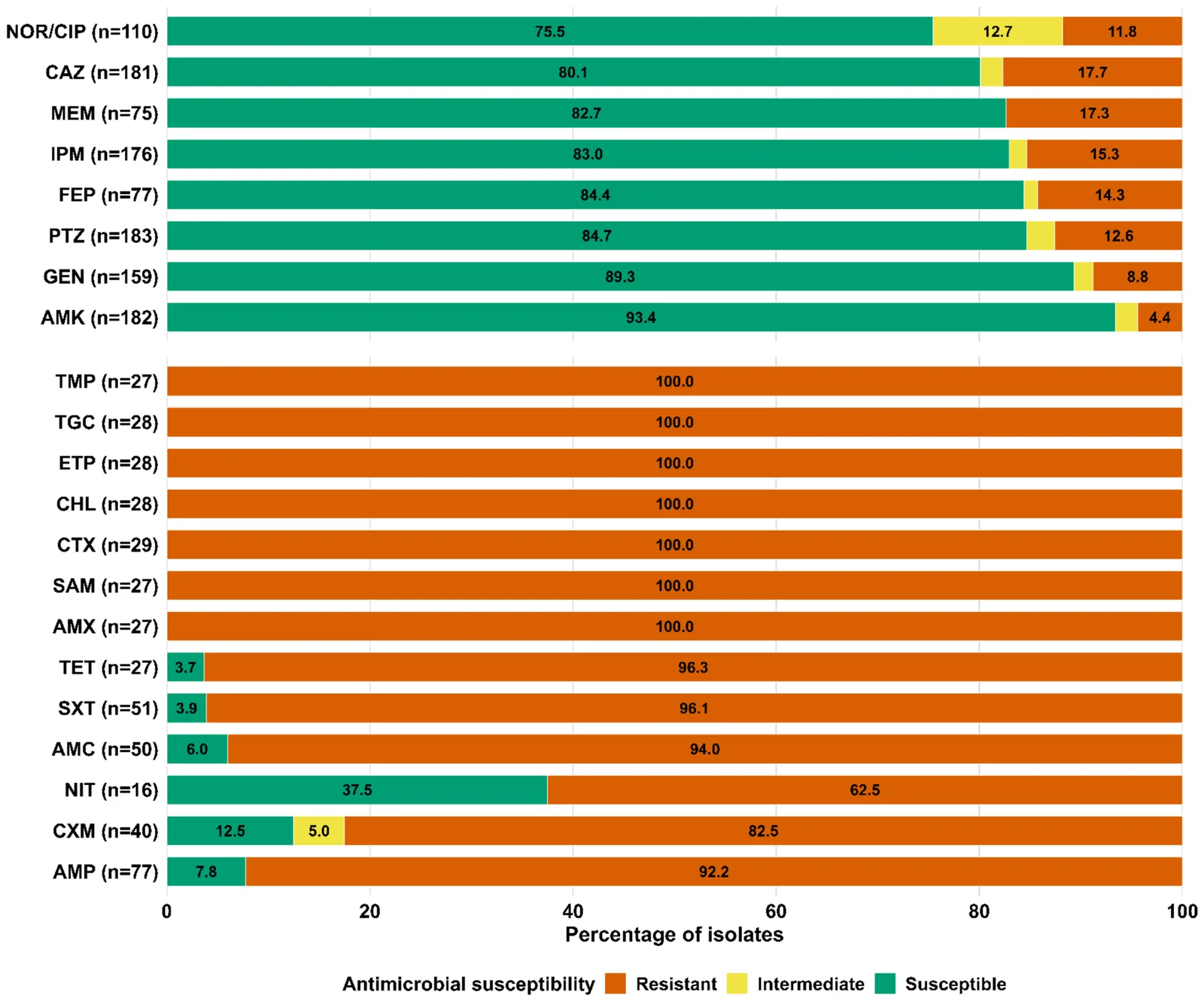
Antimicrobial Susceptibility Profile (AMK, amikacin; GEN, gentamicin; NOR/CIP, norfloxacin/ciprofloxacin; PTZ, piperacillin/tazobactam; FEP, cefepime; IPM, imipenem; MEM, meropenem; CAZ, ceftazidime; NIT, nitrofurantoin; CXM, cefuroxime; AMP, ampicillin; AMC, amoxicillin/clavulanic acid; TET, tetracycline; AMX, amoxicillin; SAM, amoxicillin/sulbactam; CTX, cefotaxime; CHL, chloramphenicol; ETP, ertapenem; TGC, tigecycline; TMP, trimethoprim; SXT, trimethoprim/sulfamethoxazole). *n* indicates the number of *Pseudomonas aeruginosa* isolates tested for each antimicrobial agent. Percentages represent the proportion of isolates classified as susceptible, intermediate, or resistant among those tested. Agents with intrinsic resistance or limited/non-applicable activity against *P. aeruginosa* are shown for completeness and should not be interpreted as evidence of acquired antimicrobial resistance.

The antimicrobial susceptibility pattern varied across the tested antibiotics (Figure 1), with the number of isolates tested differing between antimicrobial agents. Amikacin showed the highest susceptibility (170/182, 93.4%), followed by gentamicin (142/159, 89.3%), piperacillin/tazobactam (155/183, 84.7%), cefepime (65/77, 84.4%), imipenem (146/176, 83.0%), meropenem (62/75, 82.7%), and ceftazidime (145/181, 80.1%). No susceptible isolates were identified for amoxicillin, amoxicillin/sulbactam, cefotaxime, chloramphenicol, ertapenem, tigecycline, or trimethoprim. Intermediate susceptibility was uncommon across the tested antibiotics.

### 3.3. Diagnosis-Specific Antimicrobial Resistance Patterns of Pseudomonas aeruginosa

**Figure 2:**
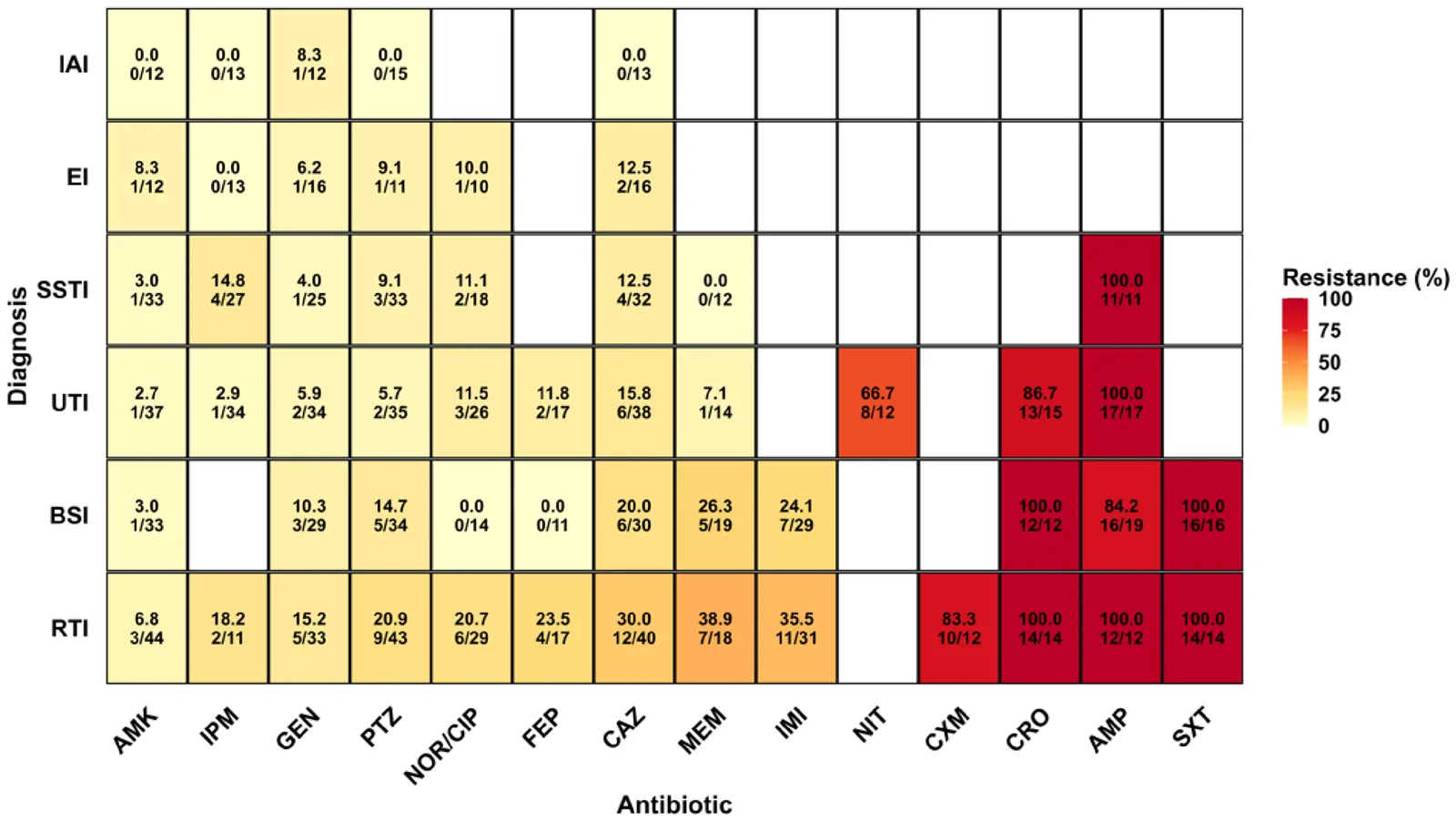
Antimicrobial Resistance Profiles Based on Diagnosis (AMK, amikacin; GEN, gentamicin; NOR/CIP, norfloxacin/ciprofloxacin; PTZ, piperacillin/tazobactam; FEP, cefepime; IPM, imipenem; MEM, meropenem; CAZ, ceftazidime; NIT, nitrofurantoin; CXM, cefuroxime; AMP, ampicillin; SXT, trimethoprim/sulfamethoxazole).

There were variations in resistance patterns according to the type of infection (Figure 2). Resistance patterns in respiratory tract and blood stream infections were generally higher compared to other types of infections, particularly to carbapenems, cephalosporins, and piperacillin/tazobactam. In bloodstream infections, resistance to ceftriaxone and trimethoprim/sulfamethoxazole reached 100%, whereas amikacin, norfloxacin/ciprofloxacin and cefepime remained highly active, with resistance rates of only 3.0% and 0%, respectively. Similarly, respiratory tract infections showed 100% resistance against ampicillin, ceftriaxone, and trimethoprim/sulfamethoxazole, together with relatively high resistance to meropenem (38.9%) and imipenem (35.5%). In contrast, urinary tract, skin and soft tissue, eye, and intra-abdominal infections demonstrated comparatively lower resistance to key antipseudomonal agents, particularly amikacin, gentamicin, imipenem, ceftazidime, and piperacillin/tazobactam.

### 3.4. Univariable Analysis of Factors Associated with In-Hospital Mortality

Univariable logistic regression analysis was performed to assess the association between individual demographic and clinical factors and in-hospital mortality. Higher odds of mortality were observed among patients admitted to the NICU/neonatology ward (UOR = 9.36, 95% CI: 3.72–23.59; *p* < 0.001), those with bloodstream infection (UOR = 7.44, 95% CI: 3.01–18.35; *p* < 0.001), blood specimens (UOR = 5.10, 95% CI: 2.02–12.87; *p* = 0.001), and ICU admission (UOR = 3.81, 95% CI: 1.45–10.02; *p* = 0.009). Pediatric status was also associated with mortality (UOR = 10.84, 95% CI: 0.64–182.96; *p* = 0.017), although the confidence interval was wide (Table 3).

### 3.5. Multivariable Analysis of Factors Associated with In-Hospital Mortality

Multivariable logistic regression identified NICU/neonatology admission and bloodstream infection as independent predictors of in-hospital mortality (Table 4). Among the 198 patients, 25 mortality events were observed, and the final Firth penalized logistic regression model included three predictors selected based on clinical relevance and available observations. Patients admitted to the NICU/neonatology ward had 7.16-fold higher odds of death compared with those admitted to other wards (AOR = 7.16, 95% CI: 2.77–19.87; *p* < 0.001). Similarly, patients with bloodstream infection had 13.20-fold higher odds of mortality than those with infections at other sites (AOR = 13.20, 95% CI: 2.25–80.44; *p* = 0.005). In contrast, blood specimens were not independently associated with mortality (AOR = 0.27, 95% CI: 0.04–1.73; *p* = 0.167). The wide confidence interval for bloodstream infection indicates substantial uncertainty in the estimated association, likely reflecting the limited number of mortality events and sparse data. Bloodstream infection and blood specimen were retained as distinct predictors because they represent different clinical variables, although substantial overlap was observed; they were not identical, as some bloodstream infections involved non-blood specimens.

## 4. Discussion

This study highlights the clinical importance of *P. aeruginosa* infections in a specialized maternity and children’s hospital, particularly among pediatric and neonatal patients. The predominance of respiratory, urinary, bloodstream, and skin and soft tissue infections is consistent with the broad clinical spectrum of *P. aeruginosa* reported in vulnerable hospitalized populations [23,26,27,28]. Its capacity to persist in hospital environments and acquire antimicrobial resistance further complicates treatment and infection control [29]. These findings underscore the importance of interpreting the local clinical profile alongside antimicrobial resistance patterns when developing infection-management and stewardship strategies.

From our antimicrobial susceptibility analysis we found that amikacin remained the most active antimicrobial agent against *P. aeruginosa*. High susceptibility was also observed for gentamicin, piperacillin/tazobactam, cefepime, imipenem, meropenem, and ceftazidime, indicating that these agents continue to retain substantial activity against clinical isolates in our institution. These findings generally agree with the recent Saudi national antimicrobial surveillance study, which also identified amikacin as one of the most active antibiotic against *P. aeruginosa*, although the susceptibility observed in our study (93.4%) was considerably higher than the national estimate of 76.8% [23,30]. A regional ATLAS surveillance study from Africa and the Middle East similarly reported amikacin as one of the most active agents against *P. aeruginosa*, supporting our findings [31]. However, susceptibility patterns may vary considerably between individual hospitals and patient populations. A multicenter study from tertiary care hospitals in Makkah and Jeddah reported differences in resistance patterns between hospitals, despite the use of the same system for identification and antimicrobial susceptibility testing, highlighting the potential influence of local hospital and patient characteristics on observed resistance rates [32]. Similarly, an 11-year study from a tertiary hospital in Makkah included predominantly older male patients and reported substantial temporal variation in resistance, with resistance rates decreasing during the later years following implementation of infection-control protocols and stricter antibiotic-prescribing policies [33]. In addition, a 10-year single-center study from a tertiary hospital in Riyadh reported carbapenem resistance of 40% and an MDR percentage of 37.5% [26]. This MDR percentage is relatively close to the 41.4% observed in our study; however, the comparison should be interpreted in the context of the study populations and clinical settings. The Riyadh study included isolates from ICUs also, whereas the King Fahad Medical City study included 3579 clinical isolates and reported that respiratory samples contained the majority of resistant isolates, with MDR and XDR strains more frequently observed among male patients [16,34]. These differences in patient characteristics, hospital setting, ICU representation, and specimen distribution may contribute to variation in reported MDR percentage. This difference may reflect variations in patient populations, hospital type, local antimicrobial stewardship practices, and regional resistance epidemiology [35,36,37].

The mortality analysis further identified clinically meaningful factors associated with poor outcomes. Admission to the NICU/neonatology ward and bloodstream infection remained independent predictors of mortality after multivariable adjustment. The association between NICU/neonatology admission and mortality should be interpreted cautiously, as ward admission may reflect a combination of patient characteristics and clinical factors, including younger age, underlying illness, disease severity, invasive procedures, and prolonged hospitalization, rather than a direct effect of ward location itself [1,20,38]. This is consistent with previous evidence showing that delayed initiation of effective antimicrobial therapy is associated with increased mortality in patients with *P. aeruginosa* bloodstream infections [39]. Similarly, previous studies have shown that severe sepsis and high-risk bloodstream infections are major independent predictors of mortality in patients with *P. aeruginosa* bacteremia [40]. The observed association between NICU/neonatology admission and mortality highlights the importance of early recognition, appropriate antipseudomonal therapy, and strengthened infection prevention measures in neonatal intensive care settings [17,21,41].

There are numerous strengths of this study. It provides one of the few detailed evaluations of *P. aeruginosa* infections in a maternity and children’s hospital in Saudi Arabia by integrating demographic characteristics, infection sites, antimicrobial susceptibility patterns, diagnosis-specific resistance profiles, and mortality risk factors within a single analysis. There are also a number of limitations that need to be addressed. Firstly, it is a retrospective single-center study, which might affect the generalizability of the findings to other healthcare settings. Secondly, antimicrobial susceptibility testing was done according to routinely available clinical laboratory data and no molecular investigation into resistance mechanisms was conducted. Future multicenter prospective studies incorporating molecular resistance profiling and larger patient populations would further improve understanding of resistance epidemiology and clinical outcomes of *P. aeruginosa* infections in Saudi Arabia. Additionally, resistance estimates for some subgroups may have been unstable because of small subgroup sample sizes and should therefore be interpreted cautiously. Another limitation was the heterogeneous documentation of comorbidities and clinical risk factors in the retrospective records. These variables were frequently recorded as free-text clinical descriptions, with multiple conditions or risk factors documented within individual records, which limited their reliable standardization and quantitative presentation in the main tables. In addition, mortality analysis may also have been affected by residual confounding, as some important clinical factors, including disease severity and treatment-related factors, were not captured in the final model. The small number of mortality events may have limited the ability to precisely estimate mortality associations. As this was a retrospective study based on clinical records, some degree of selection bias and misclassification of infection and colonization may also have occurred. Furthermore, susceptibility results were based on the available number of isolates tested for each antimicrobial agent, which varied across antibiotics. The relationship between MDR status and mortality was not specifically evaluated and should be examined in future studies with larger sample sizes and sufficient mortality events.

## 5. Conclusions

Overall, the findings indicate that while some of the commonly used antipseudomonal drugs, such as amikacin, gentamicin, cefepime, piperacillin/tazobactam, and carbapenems, remain highly active against *P. aeruginosa* infections at this hospital, presence of drug-resistant *P. aeruginosa* remains high among specific antibiotics and varies considerably according to the site of infection. These findings reinforce the need for continuous local antimicrobial resistance surveillance, diagnosis-specific antibiograms, antimicrobial stewardship programs, and the targeted infection prevention strategies to optimize patient outcomes and limit the emergence of resistant *P. aeruginosa* strains in pediatric healthcare settings.

## Figures and Tables

**Table 1:** Demographic and Clinical Characteristics of Patients (N = 198).

Characteristic	*n* (%)
Gender	
Male	107 (54.0)
Female	91 (46.0)
Age Group	
Infant (<1 year)	24 (12.1)
Pediatric child (1–10 years)	123 (62.1)
Pediatric adolescent (11–18 years)	21 (10.6)
Adults	30 (15.1)
Ward Admitted To	
NICU/neonatology	49 (24.7)
Pediatrics Specialty	33 (16.7)
ICU	26 (13.1)
General Surgery	21 (10.6)
OB-GYNE	17 (8.6)
Pediatric Cardiology	12 (6.1)
Emergency Department	9 (4.5)
Chemotherapy	7 (3.5)
Other wards	24 (12.1)
Diagnosis	
Respiratory tract infection	45 (22.7)
Urinary tract infection	40 (20.2)
Bloodstream infection	35 (17.7)
Skin and soft tissue infection	34 (17.2)
Eye infection	16 (8.1)
Intra-abdominal infection	16 (8.1)
Other diagnoses	12 (6.1)
Site of Infection	
Respiratory tract	45 (22.7)
Skin/soft tissue	42 (21.2)
Urinary tract	39 (19.7)
Bloodstream	34 (17.2)
Eye	16 (8.1)
Abdomen	15 (7.6)
Other sites	7 (3.5)
Type of Sample	
Pus	60 (30.3)
Tracheal aspirate	38 (19.2)
Urine	38 (19.2)
Blood	30 (15.2)
Eye sample	16 (8.1)
Other samples	16 (8.0)
Clinical Outcome	
Alive	173 (87.4)
Dead	25 (12.6)
MDR Status	
MDR	82 (41.4)
Non-MDR	116 (58.6)

**Table 2:** Comparison between clinical outcomes, demographic and clinical characteristics in patients infected with *Pseudomonas aeruginosa*.

Characteristic	Alive (*n* = 173) *n* (%)	Dead (*n* = 25) *n* (%)	*p*-Value
Gender			0.0867
F	84 (48.6%)	7 (28.0%)	
M	89 (51.4%)	18 (72.0%)	
Age			0.104
Adult	30 (17.4%)	0 (0.0%)	
Pediatric infant (<1 year)	18 (10.4%)	6 (24.0%)	
Pediatric adolescent (11–18 years)	20 (11.6%)	1 (4.0%)	
Pediatric child (1–10 years)	105 (60.7%)	18 (72.0%)	
Ward Admitted To			0.00232
Chemotherapy	7 (4.0%)	0 (0.0%)	
Day Care Children	3 (1.7%)	0 (0.0%)	
Day Care Female	3 (1.7%)	0 (0.0%)	
Emergency Department	9 (5.2%)	0 (0.0%)	
General Surgery	21 (12.1%)	0 (0.0%)	
High-Risk Pregnancy	1 (0.6%)	0 (0.0%)	
ICU	14 (8.1%)	4 (16.0%)	
NICU/neonatology	32 (18.5%)	17 (68.0%)	
OB-GYNE	17 (9.8%)	0 (0.0%)	
Oncology	1 (0.6%)	0 (0.0%)	
Pediatric Specialty	32 (18.5%)	1 (4.0%)	
Pediatric Cardiology	12 (6.9%)	0 (0.0%)	
Pediatric Emergency	3 (1.7%)	0 (0.0%)	
Pediatric Orthopedic	1 (0.6%)	0 (0.0%)	
Pediatric Urology Surgery	3 (1.7%)	0 (0.0%)	
Pediatrics ENT	1 (0.6%)	0 (0.0%)	
Pediatrics Infectious Diseases	1 (0.6%)	0 (0.0%)	
Pediatrics Orthopedic	1 (0.6%)	0 (0.0%)	
Pediatrics Pulmonary Diseases	2 (1.2%)	0 (0.0%)	
PICU	5 (2.9%)	3 (12.0%)	
Renal Dialysis	1 (0.6%)	0 (0.0%)	
Urogynecology	3 (1.7%)	0 (0.0%)	
Diagnosis			<0.001
Bloodstream infections	22 (12.7%)	13 (52.0%)	
Bone and joint infections	1 (0.6%)	0 (0.0%)	
CNS infection	3 (1.7%)	0 (0.0%)	
ENT infection	2 (1.2%)	0 (0.0%)	
Ethylmalonic Encephalopathy	1 (0.6%)	0 (0.0%)	
Eye infections	14 (8.1%)	2 (8.0%)	
Intra-abdominal infection	14 (8.1%)	2 (8.0%)	
Respiratory Tract infection	38 (22.0%)	7 (28.0%)	
Skin and Soft Tissue infections	33 (19.1%)	1 (4.0%)	
Skin infection	5 (2.9%)	0 (0.0%)	
Urinary tract infection	40 (23.1%)	0 (0.0%)	
Site of Infection			0.001
Abdomen	13 (7.5%)	2 (8.0%)	
Blood	23 (13.3%)	11 (44.0%)	
Bone	1 (0.6%)	0 (0.0%)	
Brain	1 (0.6%)	0 (0.0%)	
CSF	2 (1.2%)	0 (0.0%)	
Ear	2 (1.2%)	0 (0.0%)	
Eye	14 (8.1%)	2 (8.0%)	
Pus swabs from central line	1 (0.6%)	0 (0.0%)	
Respiratory tract	38 (22.0%)	7 (28.0%)	
right lower quadrant of the abdomen	1 (0.6%)	0 (0.0%)	
Sepsis wound swab	0 (0.0%)	1 (4.0%)	
Skin	27 (15.6%)	0 (0.0%)	
Skin and soft tissue infection	0 (0.0%)	1 (4.0%)	
Surgical wounds	1 (0.6%)	0 (0.0%)	
Urinary tract	39 (22.5%)	0 (0.0%)	
Vulva	1 (0.6%)	0 (0.0%)	
Wound Infection	9 (5.2%)	1 (4.0%)	

M, male; F, female. *p* < 0.05 was considered statistically significant.

**Table 3:** Univariable Logistic Regression Analysis of Factors Associated with In-Hospital Mortality.

Predictor	UOR	95% CI (LCL–UCL)	*p*-Value
Male	2.43	0.96–6.11	0.084
Pediatric	10.84	0.64–182.96	0.017
Infant (<1 year)	2.72	0.96–7.69	0.092
NICU/neonatology ward	9.36	3.72–23.59	<0.001
ICU	3.81	1.45–10.02	0.009
Bloodstream infection	7.44	3.01–18.35	<0.001
Respiratory infection	1.38	0.54–3.55	0.609
Blood specimen	5.10	2.02–12.87	0.001

UOR, unadjusted odds ratio; CI, confidence interval; NICU, neonatal intensive care unit; ICU, intensive care unit.

**Table 4:** Multivariable Logistic Regression Analysis of Factors Associated with In-Hospital Mortality among Patients with *Pseudomonas aeruginosa* Infection.

Predictor	B	SE	AOR	95% CI (LCL–UCL)	*p*-Value
NICU/neonatology ward (vs. other wards)	1.968	0.493	7.16	2.77–19.87	<0.001
Bloodstream infection (vs. other site)	2.580	0.869	13.20	2.25–80.44	0.005
Blood specimen (vs. other specimen)	−1.312	0.931	0.27	0.04–1.73	0.167

LCL, lower confidence limit; UCL, upper confidence limit. *p* < 0.05 was considered statistically significant. Relatively wide 95% confidence intervals indicate uncertainty around the estimated associations.

## Data Availability

The original contributions presented in this study are included in the article. Further inquiries can be directed to the corresponding author.

## Abbreviation

AMR	Antimicrobial resistance
AST	Antimicrobial susceptibility testing
CLSI	Clinical and Laboratory Standards Institute
Firth	Firth penalized logistic regression
ICU	Intensive Care Unit
